# Cardiovascular magnetic resonance demonstrates structural cardiac changes following transjugular intrahepatic portosystemic shunt

**DOI:** 10.1038/s41598-021-92064-8

**Published:** 2021-06-16

**Authors:** Ulf K. Radunski, Johannes Kluwe, Malte Klein, Antonio Galante, Gunnar K. Lund, Christoph Sinning, Sebastian Bohnen, Enver Tahir, Jitka Starekova, Peter Bannas, Christian Stehning, Gerhard Adam, Ansgar W. Lohse, Stefan Blankenberg, Kai Muellerleile, Daniel Benten

**Affiliations:** 1 General and Interventional Cardiology, University Heart and Vascular Center Hamburg, Martinistrasse 52, 20246 Hamburg, Germany; 2 I. Department of Medicine, Gastroenterology and Hepatology, University Medical Center Hamburg-Eppendorf, Hamburg, Germany; 3 Department of Diagnostic and Interventional Radiology and Nuclear Medicine, University Medical Center Hamburg-Eppendorf, Hamburg, Germany; 4 Philips Research Hamburg, Hamburg, Germany; 5 DZHK (German Center for Cardiovascular Research), Partner Site Hamburg/Kiel/Lübeck, Hamburg, Germany

**Keywords:** Hepatology, Liver diseases, Liver cirrhosis, Portal hypertension, Cardiology

## Abstract

Transjugular intrahepatic portosystemic shunt (TIPS) reduces portal hypertension in patients with liver cirrhosis. The exact cardiac consequences of subsequent increase of central blood volume are unknown. Cardiovascular magnetic resonance (CMR) imaging is the method of choice for quantifying cardiac volumes and ventricular function. The aim of this study was to investigate effects of TIPS on the heart using CMR, laboratory, and imaging cardiac biomarkers. 34 consecutive patients with liver cirrhosis were evaluated for TIPS. Comprehensive CMR with native T1 mapping, transthoracic echocardiography, and laboratory biomarkers were assessed before and after TIPS insertion. Follow-up (FU) CMR was obtained in 16 patients (47%) 207 (170–245) days after TIPS. From baseline (BL) to FU, a significant increase of all indexed cardiac chamber volumes was observed (all *P* < 0.05). Left ventricular (LV) end-diastolic mass index increased significantly from 45 (38–51) to 65 (51–73) g/m^2^ (*P* =  < 0.01). Biventricular systolic function, NT-proBNP, high-sensitive troponin T, and native T1 time did not differ significantly from BL to FU. No patient experienced cardiac decompensation following TIPS. In conclusion, in patients without clinically significant prior heart disease, increased cardiac preload after TIPS resulted in increased volumes of all cardiac chambers and eccentric LV hypertrophy, without leading to cardiac impairment during follow-up in this selected patient population.

## Introduction

Portal hypertension is a major complication of advanced liver cirrhosis. It may lead to systemic and splanchnic vasodilatation as well as hyperdynamic circulation, which is characterized by increased cardiac output, low systemic vascular resistance, and low arterial blood pressure^1^. Insertion of a transjugular intrahepatic portosystemic shunt (TIPS) effectively alleviates portal hypertension and is indicated in patients with refractory ascites and/or risk of recurrent variceal bleeding^2^. Beside its beneficial effects, TIPS increases cardiac preload resulting from increased shunting of blood from the splanchnic vascular bed into the central vascular bed and thereby increases the central blood volume and aggravates the hyperdynamic circulation state^3^. These changes can lead to acute heart failure following TIPS implantation in 0.9 to 20%^4,5^. Therefore, transthoracic echocardiography is routinely performed in most centers before TIPS implantation in order to detect patients at highest risk for acute heart failure after TIPS. Congestive heart failure, severe tricuspid regurgitation and severe pulmonary hypertension are considered as cardiac contraindications for TIPS implantation^2^. However, despite a careful echocardiography-based patient selection, post-TIPS heart failure still occurs in some cases. Thus, there is uncertainty in clinical routine whether more subtle cardiac phenotypes are at risk for cardiac decompensation after TIPS implantation since diastolic dysfunction, left atrial dilation, and other findings have been detected as potential predictors for acute heart failure following TIPS implantation^4–6^. To date, little is known about cardiac stress and adaptive cardiac changes after TIPS.

Cardiovascular magnetic resonance imaging (CMR) is currently the reference standard for determination of left and especially right ventricular volumes and function with high intraobserver, interobserver, and test–retest reproducibility^7^. Moreover, CMR is also the major non-invasive tool for myocardial tissue characterization^8^. In particular, T1 mapping is a novel parametric technique that permits routine quantification of changes in myocardial tissue composition, based on changes in T1 relaxation times without the need of contrast agent^8^. Multiple lines of evidence suggest a central role for T1 mapping in detection of diffuse myocardial disease even in early stages of infiltrative diseases, acute myocardial injury as well as diffuse fibrosis, which is a common finding in many cardiomyopathies^9^. To the best of our knowledge, the medium- and long-term effects of TIPS on cardiac function and volumes have not been assessed so far using CMR.

In the present study, our aim was to characterize how TIPS insertion affects cardiac function in patients with liver cirrhosis in order to identify possible mechanisms leading to post-TIPS heart failure.

## Results

### Patient characteristics at baseline

Detailed patients’ characteristics and laboratory results of all 34 patients with CMR at baseline (BL) are displayed in Supplementary Table S1. Of patients who received both, BL and follow-up (FU) CMR (N = 16; Table 1), median age was 57 (49–67) years. 8 patients (50%) were male. Etiology of liver cirrhosis was alcoholic liver disease in 69%. 94% of patients presented with Child–Pugh class B. Median Child–Pugh score was 7 (7–8) and median MELD score was 13 (11–16). 25% of patients had previous mild hepatic encephalopathy (grade 1–2) and 88% had ascites. 31% of patients had a history of previous esophageal variceal bleeding. With respect to comorbidities, 13% had coronary artery disease, 19% diabetes mellitus. 94% of all patients were treated with spironolactone with a median dose of 100 (50–100) mg. 75% of patients were treated with loop diuretics, 19% with non-selective beta-blockers and 6% with an ACE inhibitor. No patient showed impaired systolic left ventricular (LV) function or grade 3 diastolic dysfunction in TTE before TIPS and CMR, respectively.

**Table 1:** Patient characteristics of all patients with follow-up CMR (N = 16).

Age (years)	57 (49–67)
Male sex (%)	8 (50)
Body mass index (kg/m^2^)	24 (21–29)
Alcoholic liver disease (%)	11 (69)
Child–Pugh score	7 (7–8)
Child–Pugh class (%)	
A	1 (6)
B	15 (94)
C	0
MELD score	13 (11–16)
Hepatic encephalopathy grade 1–2 (%)	4 (25)
Ascites at CMR (%)	14 (88)
Previous esophageal variceal bleeding (%)	5 (31)
CAD (%)	2 (13)
Diabetes mellitus (%)	3 (19)
Medication	
Spironolactone (%)	15 (94)
Loop diuretics (%)	12 (75)
Beta blockers (%)	3 (19)
ACE inhibitor (%)	1 (6)
Laboratory parameters	
Hemoglobin (g/dl)	9.4 (8.5–11.3)
Sodium (mmol/l)	137 (132–139)
Albumin (g/l)	27 (23–31)
Bilirubin (mg/dl)	1.3 (0.9–1.5)
Urea (mg/dl)	25 (19–31)
Creatinine (mg/dl)	1.4 (1.1–1.7)
Glomerular filtration rate (ml/min)	45 (41–60)
AST (U/l)	37 (30–50)
ALT (U/l)	21 (13–25)
GGT (U/l)	132 (77–209)
Creatin kinase (U/l)	63 (27–82)
hs-TnT (pg/ml)	19 (13–28)
NT-proBNP (ng/l)	215 (174–497)

Values are median (interquartile range) or n (%).

ACE inhibitor = angiotensin-converting-enzyme inhibitor; AST = Aspartate Aminotransferase; ALT = Alanine Aminotransferase; GGT = Gamma-Glutamyl Transferase; BMI = body mass index; CAD = coronary artery disease; CMR = cardiovascular magnetic resonance imaging; hs-TnT = high-sensitive Troponin T; MELD score = Model for End-stage Liver Disease score; NT-proBNP = N-terminal pro-B-type natriuretic peptide.

### Patients’ individual outcome

**Figure 1:**
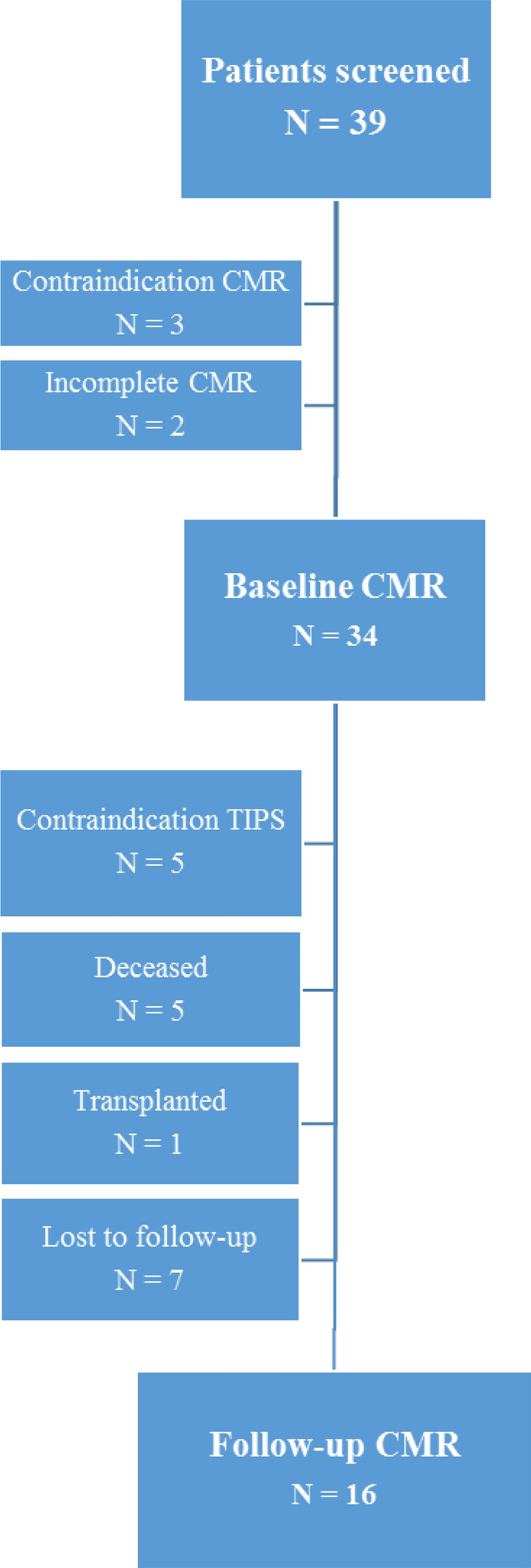
Flow chart of prospective consecutive screening, inclusion and exclusion of patients.

We prospectively screened 39 patients with liver cirrhosis and refractory ascites who were evaluated for TIPS implantation in our tertiary care center. The only exclusion criteria were contraindications for CMR. Three of 39 had to be excluded due to contraindication for CMR and in two patients CMR had to be interrupted due to unknown claustrophobia (Fig. 1). Of 34 patients with CMR at BL, 13 did not receive a FU CMR: Seven patients were lost to FU, one patient received liver transplantation, and five died before scheduled FU CMR. The causes of death were sepsis (N = 2), hemorrhagic shock after TIPS implantation (N = 1) and acute-on-chronic liver failure (N = 2) (Fig. 1). Median time to FU CMR was 207 (170–245) days after TIPS insertion. In five patients TIPS was not performed due to relative or absolute contraindications such as overt hepatic encephalopathy (N = 1), medically treatable ascites (N = 1), not significantly increased portosystemic gradient (N = 1), relevant tricuspid with mitral regurgitation or aortic valve stenosis (N = 2). None of our patients (N = 34) experienced acute heart failure defined as rapid development or change of symptoms and signs of heart failure that requires urgent medical attention^14^. Five patients showed an increase of NT-proBNP > 50% despite preserved LV function at BL and FU. Two of these five patients presented with diastolic dysfunction (DD) already at BL, and two patients showed a newly occurred DD or progression of initially existing DD, respectively. Following TIPS implantation, median portosystemic pressure gradient decreased from 21 ± 3 to 7 ± 3 mmHg, indicating adequate function of implanted stents.

### Cardiac mass, function and volumes before and after TIPS implantation

**Figure 2:**
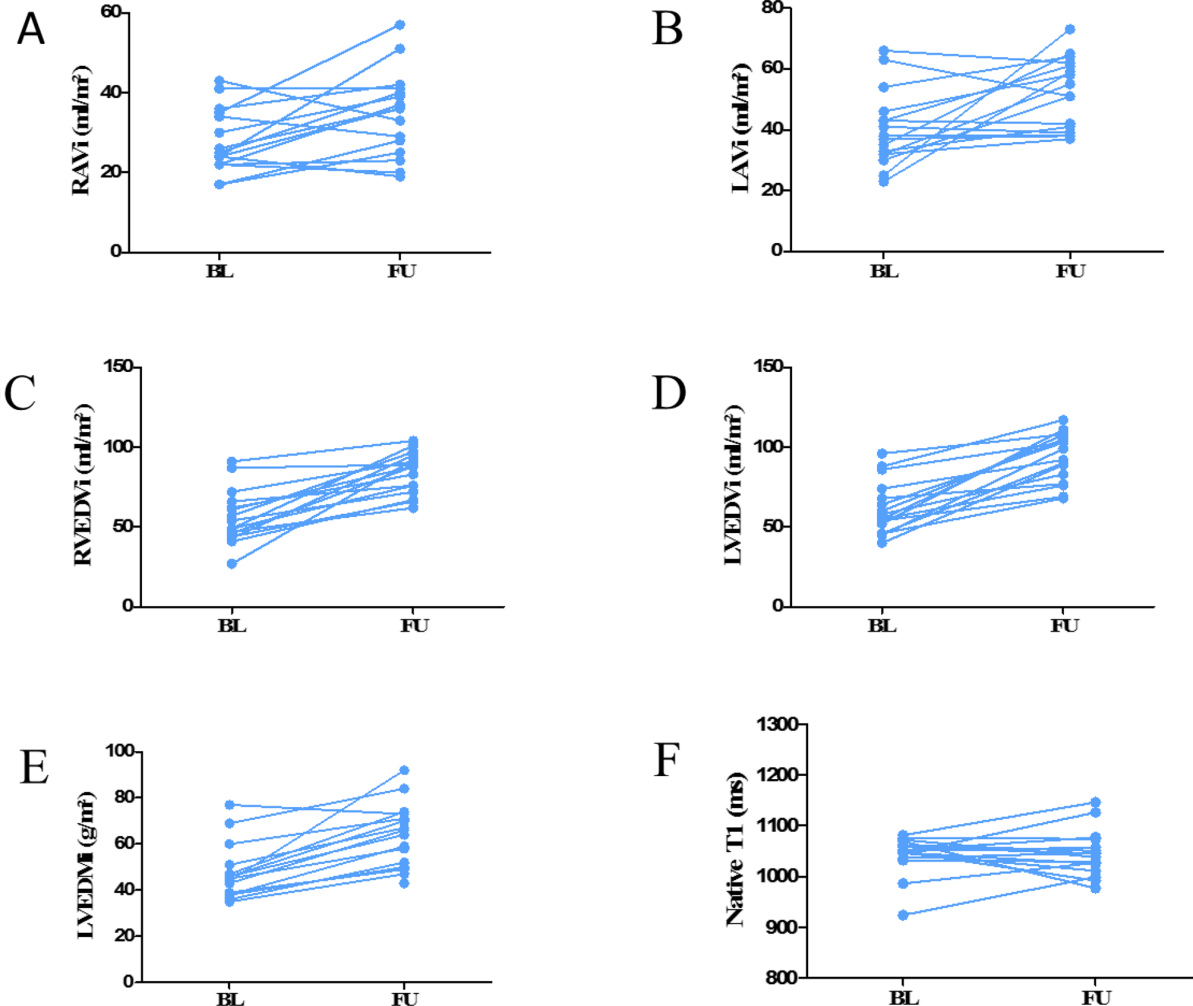
Individual development of RAVi (**A**), LAVi (**B**), RVEDVi (**C**), LVEDVi (**D**), LVEDMi (**E**) and global native T1 (**F**) from BL to FU. Created with: GraphPad Prism 6.00; https://www.graphpad.com/scientific-software/prism/. *Abbreviations*: LAVi = indexed left atrial volume; LVEDMi = indexed left ventricular end-diastolic mass; LVEDVi = indexed left ventricular end-diastolic volume; RAVi = indexed right atrial volume; RVEDVi = indexed right ventricular end-diastolic volume.

CMR results of all 34 cirrhotic patients at the time of TIPS evaluation are displayed in Supplementary Table S2. Differences of CMR variables before and after TIPS implantation (N = 16) are shown in Table 2. We observed a significant increase of volumes in all cardiac chambers after TIPS implantation (Fig. 2). The median right atrial volume index (RAVi) increased from 25 (22–35) to 36 (26–41) ml/m^2^ (*P* = 0.01) and median left atrial volume index (LAVi) from 38 (32–45) to 53 (40–62) ml/m^2^ (*P* < 0.01). The median right ventricular end-diastolic volume (RVEDVi) increased from 52 (45–65) to 88 (73–94) ml/m^2^ (*P* < 0.01), median right ventricular end-systolic volume (RVESVi) from 17 (15–24) to 24 (19–37) ml/m^2^ (*P* < 0.01) and median right ventricular stroke volume (RVSVi) from 35 (31–45) to 56 (52–65) ml/m^2^ (*P* < 0.01). The median right ventricular ejection fraction (RVEF) showed no significant change from BL (67 (65–69) %) to FU (70 (63–76) %; *P* = 0.25).

**Table 2:** CMR variables, TTE variables, and cardiovascular biomarkers of all patients with both, baseline and follow-up CMR (N = 16).

CMR variables	Baseline	Follow-up	*P* value
**RAVi** (ml/m^2^)	25 (22–35)	36 (26–41)	**0.01**
**LAVi** (ml/m^2^)	38 (32–45)	53 (40–62)	**< 0.01**
**RVEDVi** (ml/m^2^)	52 (45–65)	88 (73–94)	**< 0.01**
**RVESVi** (ml/m^2^)	17 (15–24)	24 (19–37)	**< 0.01**
**RVSVi** (ml/m^2^)	35 (31–45)	56 (52–65)	**< 0.01**
**RVEF** (%)	67 (65–69)	70 (63–76)	0.25
**LVEDVi** (ml/m^2^)	57 (48–73)	96 (79–107)	**< 0.01**
**LVESVi** (ml/m^2^)	14 (12–19)	25 (16–32)	**< 0.01**
**LVSVi** (ml/m^2^)	47 (37–57)	73 (62–80)	**< 0.01**
**LVEF** (%)	75 (67–81)	76 (70–79)	0.73
**LVEDMi** (g/m^2^)	45 (38–51)	65 (51–73)	**< 0.01**
**Global native T1** (ms)	1049 (1038–1067)	1042 (1015–1070)	1.0
**Cardiovascular biomarkers**			
**NT-proBNP (ng/l)**	215 (174–497)	218 (136–367)	0.50
**hs-TnT (pg/ml)**	19 (13–28)	18 (11–40)	0.91
**TTE variables**			
**RA area (cm**^**2**^**)**	16 (13–19)	18 (15–23)	**< 0.01**
**LAVi (ml/m**^**2**^**)**	23 (20–27)	32 (28–44)	**0.02**
**TAPSE (mm)**	26 (22–33)	28 (26–34)	0.12
**LVEF (%)**	64 (60–72)	64 (60–69)	0.54
**E/e**′	8 (5–10)	11 (8–15)	**0.01**
**Diastolic dysfunction (%)**			
grade 0	13 (81)	11 (69)	–
grade 1	2 (13)	2 (13)	–
grade 2	1 (6)	3 (19)	–
grade 3	0	0	–
any	3 (19)	5 (31)	–
Progression grade 1 – > 2	–	2	–
Stable diast. dysfunction	–	1	–
New diastolic dysfunction	–	2	–

Values are median (interquartile range) or n (%).

LAVi = indexed left atrial volume; LVEDMi = indexed left ventricular myocardial mass; LVEDVi = indexed left ventricular end-diastolic volume; LVEF = left ventricular ejection fraction; LVESVi = indexed left ventricular end-systolic volume; LVSVi = indexed left ventricular stroke volume; hs-TnT = high-sensitive Troponin T; NT-proBNP = N-terminale pro-B-type brain natriuretic peptide; RA area = right atrial area; RAVi = indexed right atrial volume; RVEF = right ventricular ejection fraction; RVEDVi = indexed right ventricular end-diastolic volume; RVSVi = indexed right ventricular stroke volume; RVESVi = indexed right ventricular end-systolic volume; TAPSE = tricuspid annular plane systolic excursion.

Concordant with the changes of RV volumes, median left ventricular end-diastolic volume (LVEDVi) significantly increased from 57 (48–73) to 96 (79–107) ml/m^2^ (*P* < 0.01), median left ventricular end-systolic volume (LVESVi) from 14 (12–19) to 25 (16–32) ml/m^2^ (*P* < 0.01) and median left ventricular stroke volume (LVSVi) from 47 (37–57) to 73 (62–80) ml/m^2^ (*P* < 0.01). The median left ventricular ejection fraction (LVEF) remained unchanged from BL (75 (67–81) %) to FU (76 (70–79) %; *P* = 0.73). Similar to the observed increase in cardiac volumes, median left ventricular myocardial mass (LVEDMi) increased from BL (45 (38–51) g/m^2^) to FU (65 (51–73) g/m^2^; *P* < 0.01).

### Laboratory and imaging cardiovascular biomarkers for heart failure, myocardial injury and diffuse fibrosis

Median NT-proBNP was slightly increased at BL (215 (174–497) ng/l; normal < 194) but showed no further increase at FU (218 (136–367) ng/l; *P* = 0.50). Median high-sensitive Troponin T (hs-TnT) was 19 (13–28) pg/ml (normal: < 14 pg/ml) at BL and showed no increase at FU (18 (11–40) pg/ml; *P* = 0.91). Global myocardial native T1 showed no significant difference from BL (1049 (1038–1067) ms) to FU (1042 (1015–1070) ms; *P* = 1.0) (Table 2; Fig. 2).

### Assessment of diastolic dysfunction using transthoracic echocardiography

TTE results of all patients with BL CMR (n = 34) are shown in Supplementary Table S2. At BL, three of 34 patients presented with diastolic dysfunction (DD) grade 1 and two patients with DD grade 2. In one of those patients with DD grade 2, TIPS was not performed due to medically treatable ascites. Differences of TTE variables and course of DD of all patients with FU CMR (N = 16) are shown in Table 2. At BL, three of 16 patients presented with DD. At FU, five patients presented with DD. Two patients showed a progression of DD grade 1 to grade 2, two presented with newly diagnosed DD grade 1, and one patient showed no progression of his stable DD grade 2 from BL to FU. The median septal and lateral E/e’ significantly increased from 8 (6–10) to 11 (8–15); *P* = 0.01), whereas median TAPSE did not differ significantly from BL to FU (25 (21–31) vs. 28 (26–34) mm; *P* = 0.12). Both, the median LAVi (23 (22–33) vs. 32 (28–44) ml/m^2^; *P* = 0.02) and the median RA area (16 (13–19) vs. 18 (15–23) cm^2^; *P* =  < 0.01) increased significantly from BL to FU (Table 2).

## Discussion

In this study, we investigated the medium-term cardiac effects resulting from hemodynamic changes after TIPS implantation using CMR and cardiovascular biomarkers. Our three major findings were: Firstly, TIPS implantation led to size increase of all four cardiac chambers. Secondly, this increase of cardiac chamber volumes was accompanied by an increase of LV myocardial mass in terms of an eccentric LV hypertrophy. Thirdly, stable right and left ventricular function as well as stable laboratory and imaging biomarkers such as NT-proBNP, hs-TnT, and myocardial native T1 time do not indicate that these cardiac adaptions caused severe myocardial injury with impairment of systolic function in patients with normal LV function, no relevant heart valve defects, and a maximum diastolic dysfunction of grade 2. None of the patients in our cohort experienced acute heart failure defined as rapid development or change of symptoms and signs of heart failure that requires urgent medical attention^14^.

In our cohort, the increased preload after TIPS induced a significant increase of all cardiac chamber volumes after a median FU of seven months. These results are in line with those of Busk et al. who evaluated 25 patients one week and four months after TIPS by assessment of systemic, cardiac, and splanchnic hemodynamics using catheterization, TTE, and cardiac biomarkers to evaluate cardiac function over time. 2D-TTE and M-Mode revealed an increase of LV internal diameter as well as an increase of LA volume from BL to FU, respectively^15^. Interestingly, we found a significant increase of myocardial mass after TIPS. Both studies used different modalities for the evaluation of LV myocardial mass and the unequal observations may be explained by the longer FU period in our study, if LV hypertrophy only arises with greater latency, and different sensitivities of TTE and CMR to detect LV hypertrophy. Mechanical overload in general can lead to LV hypertrophy and can be classified into pressure overload and volume overload, causing concentric and eccentric cardiac hypertrophy, respectively^16^. Moreover, it has been shown that the physiological volume overload during pregnancy leads to reversible LV hypertrophy^17^. Our results demonstrate an adaptive eccentric LV hypertrophy as a medium-term effect possibly preventing cardiac decompensation after TIPS.

Despite these effects, the median right and left ventricular systolic function was preserved from BL to FU. Interestingly, median NT-proBNP was slightly increased at BL, but showed no further increase at FU, which could be related to an already existing hyperdynamic circulation at BL. Hs-TnT was not increased at BL or FU. The native T1 time, which has been shown to enable the detection of diffuse myocardial disease even in early stages of in many cardiomyopathies^9^, was not increased at BL or FU. Thus, the observed hemodynamic changes and cardiac adaptions neither led to any impairment of systolic function, nor to myocardial injury or myocardial fibrosis and these observations are in line with those of Busk et al. who also found a preserved LVEF and TAPSE as well as stable values for proANP and hs-TnT four months after TIPS^15^. On the other hand, Jansen et al. have recently shown in a large retrospective study that even in patients with normal EF there may be subgroups with better contractility, as measured by LV global longitudinal strain using TTE, who experience better outcome after TIPS, with reduced rates of acute-on-chronic liver failure and mortality^18^.

Until recently there was a lack of data regarding incidence and predictors of acute heart failure after TIPS. Modha et al. found symptomatic heart failure after TIPS in eight of 883 patients (0.9%). Patients with acute heart failure showed higher pre-TIPS right atrial and portal vein pressures, higher albumin, and higher prothrombin time^5^. In contrast to this low incidence of heart failure, a current study found acute heart failure in 20% of patients following TIPS^4^. Identified risk factors for acute heart failure were prolonged QTc interval, elevated BNP or NT-proBNP, elevated E/A ratio and E/e' ratio, left atrial dilatation, and aortic stenosis. A level of BNP < 40 pg/mL and a NT-proBNP < 125 pg/mL as well the absence of DD criteria at echocardiography ruled out the risk of subsequent cardiac decompensation^4^. Especially the role of DD is under debate. Caused by stiffness of the myocardium, DD might impair the cardiac ability to handle an increased preload. Moreover, DD has been shown to be a possible predictor of death in patients with cirrhosis who are treated with TIPS^4,6^. In our cohort, only five of 34 patients presented with any DD at BL. None of these patients, even one patient with DD grade 2, experienced acute heart failure after TIPS. Nonetheless, we observed a significant increase of median E/e′ in our cohort in line with the observations of other groups^4,6^. However, the diagnosis of acute heart failure in patients with liver cirrhosis is challenging due to an overlap of clinical symptoms (e.g. dyspnea, ascites, increase in weight, and fatigue), reflected by substantially varying incidence in previous studies^4,5^. Finally, we were not able to identify possible predictors for cardiac decompensation in our small cohort of patients due to a lack of heart failure events. Our results underline that the probability of systolic heart failure after TIPS is very low in patients with preserved ejection fraction and no relevant heart valve defects or severe diastolic dysfunction. Whether CMR in addition to routinely performed TTE may add prognostic information about acute heart failure after TIPS cannot be answered. Since more possible laboratory and echocardiographic risk factors for cardiac decompensation have recently been found by Billey et al.^4^, a correlation of these findings with CMR parameters would be highly interesting. Therefore, future studies with higher numbers of patients and precise definition of acute heart failure in patients with liver cirrhosis are warranted to identify predictors of insufficient cardiac adaption as risk factor of post-TIPS heart failure.

Thought as a pilot study, the sample size is small. Many patients were referred for TIPS implantation from secondary care centers so that FU of patients was hindered, which is reflected by a relatively high rate of patients who were lost to FU. We also cannot exclude that a selection bias has influenced our findings. Patients considered for TIPS in secondary care centers could have been not referred due to external assessment of cardiac function with detection of severe systolic or diastolic impairment.

In conclusion, in patients without clinically significant prior heart disease, the increased cardiac preload after TIPS insertion resulted in increased volumes of all cardiac chambers and eccentric LV hypertrophy. However, in this highly selected patient cohort the morphologic adaptions did not lead to cardiac impairment which is reflected by a stable biventricular systolic function and stable laboratory and imaging biomarkers for heart failure, myocardial injury, and fibrosis during the follow-up period.

## Methods

### Patients and TIPS procedures

This study has been approved by the licensing ethics committee (Ärztekammer Hamburg, protocol no. PV5169) and all patients gave their written informed consent. Methods were carried out in accordance with relevant guidelines and regulations. Planned as an observational pilot-study, 39 patients with liver cirrhosis and refractory ascites who were evaluated for TIPS implantation in the Department of Gastroenterology and Hepatology at University Medical Center Hamburg-Eppendorf (UKE) from March 2016 until December 2017. Exclusion criteria were contraindications for CMR. 34 patients were scanned at BL. Median time from BL CMR to TIPS implantation was 1.5 (1.0–6.3) days. TIPS implantation was carried out as described in detail previously^10^. 16 of these 34 patients received a FU CMR after a median interval of 7.4 (5.3–8.6) months from BL CMR.

### Cardiovascular magnetic resonance imaging protocol

**Figure 3:**
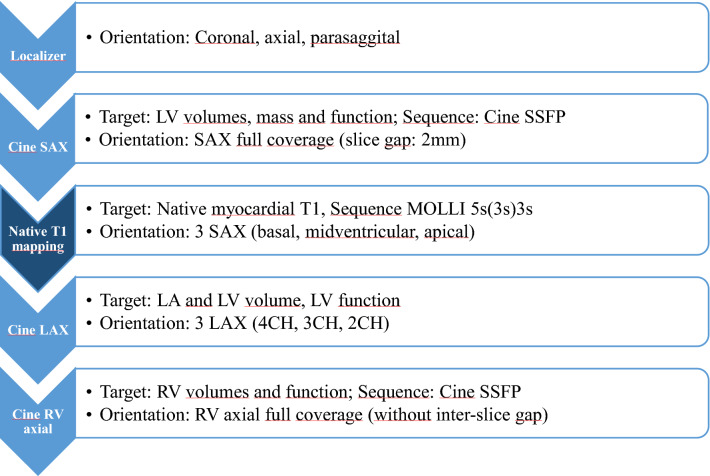
CMR protocol. *Abbreviations*: LAX = long axis view, LGE = late gadolinium enhancement, MOLLI = modified Look-Locker inversion recovery, SSFP = steady state free precession, 4CH = 4 chamber view, 3CH = 3 chamber view, 2CH = 2 chamber view.

CMR was performed on a 1.5T scanner (Achieva, Philips Medical Systems, Best, The Netherlands). The CMR protocol consisted of a standard cine-CMR with long- (four-, three-, and two-chamber view) and short-axis views as well as one axial stack for the assessment of cardiac volumes, mass, and function using a standard steady-state free-precession (SSFP) sequence. T1 mapping was performed using a 5s(3s)3s-MOLLI on 3 representative short axis slices (basal, midventricular and apical). Imaging parameters of T1 mapping were as follows: Voxel size 2 × 2 × 10 mm^3^, echo time = 0.7 ms, time to repetition = 2.3 ms, partial echo factor = 0.8, flip angle = 35°, SENSE factor = 2, linear phase encoding, ten start-up cycles to approach steady-state prior to imaging, effective inversion times between 134 and 5627 ms. The CMR protocol with typical imaging parameters is presented in Fig. 3.

### Cardiovascular magnetic resonance imaging analyses

All CMR scans were analyzed using the commercially available software cvi42 (Circle Cardiovascular Imaging Inc.). CMR measurements were performed according current guidelines^11^. LV end-diastolic and end-systolic volumes were obtained from cine-CMR short-axes to calculate LV stroke volume index (LVSVi) as well as LV ejection fraction (LVEF). LV end-diastolic mass index (LVEDMi) was obtained including papillary muscles. RV end-diastolic and end-systolic volumes were obtained from axial cine-CMR short-axes to calculate RV stroke volume index (RVSVi) as well as RV ejection fraction (RVEF). For the analysis of global native T1, endo- and epicardial contours were drawn, manually corrected for motion and carefully aligned with the contours in one basal, midventricular, and apical slice.

### Transthoracic echocardiography

All patients were examined using two-dimensional transthoracic echocardiography (TTE) before and after TIPS. Echocardiography was obtained with a Philips iE33 and EPIQ 7 (Philips Healthcare) and analyzed by two experienced cardiologists using TOMTEC ARENA Version 4.6 (TOMTEC Imaging Systems GmbH). All measurements for the assessment of RV and LV systolic function, LA and LV volumes as well as measurements for diastolic function were performed and graded according to current guidelines^12,13^.

### Statistical analyses

Statistical analysis was performed using GraphPad Prism version 6.00 (GraphPad Software, San Diego, CA, USA). Descriptive statistics were presented as median and interquartile range (Q1–Q3) and compared by Mann–Whitney U test for continuous data. Categorical data are presented as numbers and percentage and compared using Fisher’s exact test. BL and FU data was compared using paired t-test or Wilcoxon matched pairs test when the variables were not normally distributed. Two-sided *P* values were calculated and statistical significance was defined as *P* < 0.05.

## Supplementary Information

Supplementary Information.

## Data Availability

The datasets generated during and/or analyzed during the current study are available from the corresponding author on reasonable request.
