# Supplementary material for: Cardiovascular magnetic resonance demonstrates structural cardiac changes following transjugular intrahepatic portosystemic shunt
Source: Sci Rep. 2021 Jun 16;11:12719. doi: 10.1038/s41598-021-92064-8 (PMC8209000; doi:10.1038/s41598-021-92064-8)
Supplement: Supplementary file 1 — Supplementary Information. [file 41598_2021_92064_MOESM1_ESM.docx]

**Supplementary Information**

**Supplementary Table S1** Patient characteristics of all patients with CMR at baseline (N=34).

| **Age (years)** | 62 (52-66) |
| --- | --- |
| **Male sex (%)** | 22 (65) |
| **Body mass index (kg/m²)** | 24 (21-29) |
| **Alcoholic liver disease (%)** | 23 (68) |
| **Child-Pugh score** | 7 (7-8) |
| **Child-Pugh class (%)**  A  B  C | 3 (9)  31 (91)  0 |
| **MELD score** | 11.0 (9-15) |
| **Hepatic encephalopathy grade 1-2 (%)** | 9 (26) |
| **Ascites at CMR (%)** | 28 (82) |
| **Previous esophageal variceal bleeding (%)** | 8 (24) |
| **CAD (%)** | 3 (9) |
| **Diabetes mellitus (%)** | 8 (24) |
| **Terminal renal failure (%)** | 1 (3) |
| **Medication**  Spironolactone (%)  Loop diuretics (%)  Beta blockers (%)  ACE inhibitor (%) | 30 (88)  28 (82)  15 (44)  4 (12) |
| **Laboratory parameters**  Hemoglobin (g/dl)  Sodium (mmol/l)  Albumin (g/l)  Bilirubin (mg/dl)  Urea (mg/dl)  Creatinine (mg/dl)  Glomerular filtration rate (ml/min)  AST (U/l)  ALT (U/l)  GGT (U/l)  Creatin kinase (U/l)  hs-TnT (pg/ml)  NT-proBNP (ng/l) | 9.3 (8.3-11.6)  137 (132-139)  27 (23-30)  1.2 (0.7-1.5)  25 (19-31)  1.3 (1.0-1.5)  59 (42-60)  36 (28-47)  21 (14-30)  138 (77-230)  58 (41-84)  20 (13-34)  291 (189-727) |

Values are median (interquartile range) or n (%)

ACE inhibitor = angiotensin-converting-enzyme inhibitor; AST = Aspartate Aminotransferase; ALT = Alanine Aminotransferase; GGT = Gamma-Glutamyl Transferase; BMI = body mass index; CAD = coronary artery disease; CMR = cardiovascular magnetic resonance imaging; hs-TnT = high-sensitive Troponin T; MELD score = Model for End-stage Liver Disease score; NT-proBNP = N-terminal pro-B-type natriuretic peptide

**Supplementary Table S2** CMR and TTE variables of all patients with CMR at baseline (N=34).

| **CMR variables** | |
| --- | --- |
| **RAVi** (ml/m^2^) | 25 (21-37) |
| **LAVi** (ml/m^2^) | 39 (32-51) |
| **RVEDVi** (ml/m^2^) | 54 (44-63) |
| **RVESVi** (ml/m^2^) | 17 (15-24) |
| **RVSVi** (ml/m^2^) | 36 (32-43) |
| **RVEF** (%) | 67 (62-70) |
| **LVEDVi** (ml/m^2^) | 60 (53-72) |
| **LVESVi** (ml/m^2^) | 15 (12-19) |
| **LVSVi** (ml/m^2^) | 44 (39-56) |
| **LVEF** (ml/m^2^) | 75 (69-79) |
| **LVEDMi** (g/m^2^) | 46 (39-63) |
| **Global native T1** (ms) | 1049 (1036-1063) |
| **TTE variables** | |
| **RA area** (cm²) | 16 (14-21) |
| **LAVi** (ml/m²) | 27 (23-37) |
| **TAPSE** (mm) | 25 (21-31) |
| **LVEF** (%) | 65 (62-70) |
| **E/é** | 8 (6-10) |
| **Diastolic dysfunction (%)**  grade 0  grade 1  grade 2  grade 3 | 29 (85)  3 (9)  2 (6)  0 |

Values are median (interquartile range) or n (%)

LAVi = indexed left atrial volume; LVEDMi = indexed left ventricular myocardial mass; LVEDVi = indexed left ventricular end-diastolic volume; LVEF = left ventricular ejection fraction; LVESVi = indexed left ventricular end-systolic volume; LVSVi = indexed left ventricular stroke volume; RA area = right atrial area; RAVi = indexed right atrial volume; RVEF = right ventricular ejection fraction; RVEDVi = indexed right ventricular end-diastolic volume; RVSVi = indexed right ventricular stroke volume; RVESVi = indexed right ventricular end-systolic volume; TAPSE **=** tricuspid annular plane systolic excursion
